# Circulating MiR-17-5p, MiR-126-5p and MiR-145-3p Are Novel Biomarkers for Diagnosis of Acute Myocardial Infarction

**DOI:** 10.3389/fphys.2019.00123

**Published:** 2019-02-18

**Authors:** Sheng Xue, Dacheng Liu, Wenjie Zhu, Zhe Su, Liwei Zhang, Changyong Zhou, Peifeng Li

**Affiliations:** ^1^Institute for Translational Medicine, College of Medicine, Qingdao University, Qingdao, China; ^2^The Affiliated Hospital of Qingdao University, Qingdao University, Qingdao, China

**Keywords:** circulating miRNA, acute myocardial infarction, percutaneous coronary intervention, biomarker, receiver operating characteristic

## Abstract

Ischemic heart disease including myocardial infarction (MI) is a major cause of mortality and morbidity worldwide. In order to manage the acute myocardial infarction (AMI) outbreaks, novel biomarkers for risk prediction are needed. Recent studies have shown that circulating microRNAs (miRNAs) are promising biomarkers for cardiovascular diseases prediction. This study aimed to determine the possibility of circulating miRNAs used as biomarkers for AMI. The dynamic expression levels of miRNAs were examined before and after percutaneous coronary intervention (PCI) in patients. Circulating miR-17-5p, miR-126-5p, and miR-145-3p were selected and validated in 29 patients with AMI and 21 matched controls by quantitative real-time PCR. The expression levels of plasma miR-17-5p, miR-126-5p, and miR-145-3p were significantly increased in AMI patients. Receiver Operating Characteristic (ROC) analysis indicated that miR-17-5p, miR-126-5p, and miR-145-3p showed considerable diagnostic efficiency for AMI. Furthermore, we demonstrated that the combination of these three miRNAs managed to provide more accurate diagnosing of AMI.

## Introduction

Acute coronary syndrome (ACS) is one of the main cause of death worldwide (Benjamin et al., 2017). ACS is an umbrella term that refers to unstable angina pectoris (UA), ST-segment elevation myocardial infarction (STEMI), and non-ST-segment elevation myocardial infarction (NSTEMI) (Ahlin et al., 2016). The diagnosis of myocardial infarction may be based on its clinical characteristics, including electrocardiographic changes, elevated myocardial necrosis, biomarkers, and imaging changes, or confirmed by pathological examination (van Rooij and Olson, 2012). STEMI and NSTEMI are clinically defined as acute myocardial infarction (AMI) because they share the release of specific myocardial necrosis markers (Di Stefano et al., 2009). Diagnosis of AMI at an early stage is crucial to preserving heart function and minimizing myocardial injury. Creatine kinase isoenzyme (CK-MB), serum myoglobin (Myo), and cardiac troponins (cTnI, cTnT) are commonly used markers of AMI (Rajappa and Sharma, 2005). Among them, cardiac troponins (cTnI, cTnT) are considered as the “gold standard” for AMI diagnosis (Jaffe et al., 2000). However, circulating cTnI and cTnT are not specific enough to discriminate AMI from other diseases, such as severe acute ischemic stroke, chronic kidney failure, and septic shock (Abbas et al., 2005; Jensen et al., 2007; Rosjo et al., 2011). Consequently, it is meaningful to explore new biomarkers with high sensitivity and specificity in early diagnosis of AMI (McCann et al., 2008).

miRNAs are a class of short (approximately 19–25 nucleotides long), single stranded, non-coding RNA molecules that regulate gene expression at the post-transcriptional level by binding to 3′untranslated region (3′UTR) of target mRNAs (Bartel, 2004; Condorelli et al., 2014). The development in genomics, especially gene-expression profiling using microarray and quantitative real-time polymerase chain reaction (qRT-PCR), have promoted the identification of novel molecular biomarkers with potential clinical values (McPherson, 2010; Ardissino et al., 2011). In recent years, miRNAs have been verified to play a critical role in the area of cardiovascular diseases by regulating cell proliferation, differentiation, survival, and migration (Small et al., 2010).

Recent studies have shown that miRNAs may also serve as circulating biomarkers for various diseases, including tumors and cardiovascular disorders, because of their remarkable stability in the blood, urine, and other fluids (Chen et al., 2008; Mitchell et al., 2008; Gupta et al., 2010; Cortez et al., 2011). Specific expression profiles of circulating miRNA have been associated with several diseases such as cancer and cardiovascular injury (Gupta et al., 2010). miRNA as biomarker in the early stage of AMI may have advantages over cTnI, because it was proven that circulating heart-specific miRNAs could be detected in plasma of all patients within 4 h of the onset of symptoms, whereas cTnI was only detected in 85% of patients at this early stage (Wang G.K. et al., 2010). These studies confirmed that the different expression of miRNA in patients provides clues and evidence for diagnostic marker (Creemers et al., 2012).

In cardiovascular disease, neoangiogenesis and intraplaque hemorrhage are the major steps in promoting plaque vulnerability (Condorelli et al., 2014). MiR-17 and miR-126 were shown to be highly enriched in endothelial cells (ECs) (Fichtlscherer et al., 2010; Zhang et al., 2016), and miR-145 was highly expressed in smooth muscle cells (Cordes et al., 2009; Elia et al., 2009). MiR-17∼92 cluster is regulated by vascular endothelial growth factor, and may play pivotal role in regulating neoangiogenesis (Suárez et al., 2008), enhancing blood vessel growth and functional recovery of ischemic tissue (Bonauer et al., 2009). MiR-126 in apoptotic bodies can be taken by vascular smooth muscle cells (VSMCs), improving the survival of VSMCs, stabilizing and decreasing the size of atherosclerotic lesions (Zernecke et al., 2009). MiR-145 can promote contractile phenotype of VSMC, reducing plaque size in aortic sinuses, increasing the fibrous cap area, reducing the necrotic core area, and increasing plaque collagen content (Lovren et al., 2012). MiR-17, miR-126 and miR-145 were reported to be closely related to coronary artery disease (CAD), and may have the potency to be developed as biomarker (Du et al., 2016; Li et al., 2016; Zhong et al., 2018). However, the plasma expression level of miR-17, miR-126, and miR-145 in AMI patients and the potential clinical significance of these three miRNAs remain largely unknown. Therefore, the aim of the present study was to identify the plasma expression levels of miR-17, miR-126, and miR-145 in AMI patients before and after percutaneous coronary intervention (PCI), and evaluate the usefulness as a biomarker for AMI detection.

## Materials and Methods

### Ethics Statement

The study was conducted according to the Declaration of Helsinki. The ethical committee of Affiliated Hospital of Qingdao University approved the study design.

### Patients

For all participants, information about their demographic characteristics and clinical biochemistry were collected by trained interviewers. All subjects or their guardians gave written informed consent to participate in this study.

The study population consisted 29 patients with AMI (15 STEMI and 14 NSTEMI) and 21 healthy adults (non-AMI) who were consecutively recruited from Affiliated Hospital of Qingdao University between 2016 and 2017. For the AMI patients, blood samples were obtained within 4 h of onset of clinical symptoms (chest pain). The inclusion criteria for AMI patients were based on the 2012 ESC/AHA/ACC guidelines (Thygesen et al., 2012): The term acute myocardial infarction (AMI) should be used when there is evidence of myocardial necrosis in a clinical setting consistent with acute myocardial ischemia. The diagnosis for AMI meets the following criteria (Gami et al., 2015): Detection of a rise and/or fall of cardiac biomarker values, preferably high-sensitivity troponin (Hs-TnT) with at least one value above the 99th percentile upper reference limit (URL), and with at least one of the followings: (1) Symptoms of ischemia: chest pain lasting longer than 30 min. (2) New or presumed new significant ST-segment-T wave (ST-T) changes or new left bundle branch block (LBBB). (3) Development of pathological Q waves in the electrocardiogram (ECG). (4) Imaging evidence of new loss of viable myocardium or new regional wall motion abnormality. (5) Identification of an intracoronary thrombus by angiography. Healthy subjects without medical history of cardiovascular diseases were selected as controls, and they were matched by age, sex, and area of residence with the patients.

The exclusion criteria were as follows: previous history of cardiac diseases (AMI, heart failure, or cardiomyopathy), known malignancy, renal insufficiency (serum creatinine concentration >133 μmol/L), renal replacement therapy, surgery, or skeletal muscle damage within the previous months, all of which might impact the expression of miRNAs. For all the patients, the time from AMI symptoms occurred to undergoing PCI was less than 4 h. Blood samples from study subjects were collected 1 h before PCI procedures and 1 h after PCI to investigate the expression level of plasma miRNAs.

### Sample Collection and Storage

About 5 ml of vein blood samples were collected from each participant in EDTA-anticoagulant tubes (Sanli, Liuyang, China) 1 h before PCI procedures and 1 h after PCI. All samples collected from each participant were centrifuged at 3,000 rpm for 10 min at 4°C, and the plasma supernatant was removed and transferred at −80°C until be used.

### Extraction of Plasma Total RNA

RNA was extracted from plasma according to the following method: the plasma (250 μL) was mixed with 750 μL TRIzol (Life Technologies, Grand Island, NY, United States), and then shaken vigorously to ensure complete dissociation of nucleoprotein complexes. Each sample was supplemented with 5 μL aliquot of 50 pM synthetic *Caenorhabditis elegans* miR-39-3p (cel-miR-39-3p) after the addition of TRIzol to normalize miRNA expression in quantitative real-time polymerase chain reaction (qRT-PCR) (Fichtlscherer et al., 2010; Niu et al., 2015). Chloroform (200 μL) was added to the mixture and shaken vigorously. After standing at room temperature (10 min), the mixture was centrifuged at 12,000 × *g* for 10 min at 4°C. The supernatant was transferred to a new tube and mixed with 600 μL cold isopropanol. Glycogen (Thermo Fisher Scientific, Waltham, MA, United States) was used to increase the RNA yield and the solution was precipitated at −20°C overnight. All samples were then centrifuged at 12,000 *g* for 10 min at 4°C, and the supernatants were discarded. After washing with 75% ethanol twice, RNA pellet was finally dissolved by adding DEPC H_2_O (10 μL), and stored at −80°C until use. The concentration and quality of RNA were measured by NanoDrop spectrophotometer (Thermo Fisher Scientific, Waltham, MA, United States).

### MicroRNA Polyadenylation and Reverse Transcription

The cDNA was prepared using Mir-X^TM^ miRNA First Strand Synthesis Kit (Clontech Laboratories, Mountain View, CA, United States) according to the manufacture’s protocol. The reaction was performed in a thermocycler with the following program: incubation at 37°C for 1 h, then termination at 85°C for 5 min to inactivate the enzymes. Finally, an aliquot of 90 μL double distilled water (ddH_2_O) was added to bring the total volume of 100 μL.

### MicroRNA Validation

The expression of the selected plasma microRNA was determined by SYBR qRT-PCR Kit (Takara, Dalian, China) according to the manufacture’s protocol. The primer sequences of miRNAs used in qRT-PCR were listed in Supplementary Table S1. The reaction was performed with the following program: 95°C for 10 s, 40 cycles of 95°C for 5 s, 60°C for 20 s, and followed by the thermal denaturing step to generate the dissociation curves to verify amplification specificity. Cel-miR-39-3p was served as the normalization control, and data were analyzed by Bio-Rad CFX Manager software (Bio-Rad, CA, United States) to obtain miRNAs relative expression scores. Cycle threshold (Ct) values of each miRNA were normalized to cel-miR-39-3p, and the 2^−ΔΔCt^ method was used to analyze the relative expression level of miRNA.

### Statistical Analysis

Data were presented as means ± standard deviations (SD) for quantitative variables. Mean values of quantitative variables were evaluated by Student’s *t-*test, or Mann–Whitney *U*-test when Student’s *t-*test were not satisfied. For categorical variables, differences between cases and controls were analyzed by chi square (χ^2^) test or Fisher’s exact test when necessary.

The associations of miRNA expression levels among each other and with clinical variables were analyzed by Spearman rank correlations. The combination among miRNAs were assessed using logistic regression. Receiver operating characteristic (ROC) curves and the area under the ROC curves (AUC) were performed to evaluate the diagnostic accuracy of the selected miRNAs using SigmaPlot 12.5 software (Systat Software, Inc., San Jose, CA, United States). SPSS 24.0 software (SPSS Inc., Chicago, IL, United States) was used to perform the statistical analyses. All statistical tests were two-tailed, and a value of *p* < 0.05 was considered statistically significant.

## Results

### Baseline Characteristics of the Study Population

The baseline characteristics of 29 AMI patients and 21 control subjects were summarized in Table 1. The result showed that there were statistical differences between the control subjects and AMI patients. Some of the considered clinical variables such as CK-MB, hsTNT, and NT-proBNP were markedly up-regulated in the AMI patients compared to the control subjects. For the metabolic markers such as BMI, hypertension, triglycerides, LDL cholesterol, and WBC were higher in individuals with AMI.

**Table 1:** Clinical characteristics of AMI patients and the control subjects.

Variable	AMI group (*n* = 29)	Control group (*n* = 21)	*p*-value
Male/Female (n/n)^1^	23/6	16/5	0.793
Age (years)^2^	68.0 ± 10.4	58.5 ± 14.3	0.746
BMI (kg/m^2^)^2^	26.2 ± 2.6	23.9 ± 4.2	0.010^∗∗^
Smoking status			
Current smoker (%)^1^	44.8%	42.9%	1.000
Former smoker (%)^1^	17.2%	0	0.660
Never (%)^1^	37.9%	57.1%	0.252
Hypertension (%)^1^	55.2%	23.8%	0.042^∗^
Diabetes (%)^1^	27.6%	9.5%	0.160
SBP (mmHg)^3^	128.4 ± 23.8	128.1 ± 12.2	0.887
DBP (mmHg)^3^	76.9 ± 11.1	74.8 ± 8.6	0.425
Heart rate (beats/minutes)^3^	68.1 ± 7.7	76.1 ± 7.9	0.002^∗∗^
Killip class at admission ≥ II (%)^1^	20.7	0	0.033^∗^
Blood glucose (mmol/L)^3^	6.8 ± 2.8	5.4 ± 1.9	0.058
Total cholesterol (mmol/L)^3^	4.2 ± 1.7	4.8 ± 0.9	0.031^∗^
Triglycerides (mmol/L)^3^	1.8 ± 1.4	1.1 ± 0.6	0.006^∗∗^
HDL (mmol/L)^3^	1.1 ± 0.3	1.1 ± 0.5	0.941
LDL (mmol/L)^3^	3.0 ± 0.8	2.4 ± 1.3	0.038^∗^
WBC (×10^9^/L)^3^	9.1 ± 2.9	7.1 ± 2.2	0.022^∗^
Cr (μmol/L)^3^	61.4 ± 17.7	63.9 ± 26.2	0.344
NT-proBNP (pg/ml)^3^	1022.6 ± 1366.8	107.7 ± 0.8	0.027^∗^
CK-MB (U/L)^3^	31.7 ± 33.3	18.2 ± 24.7	0.011^∗^
MYO (μg/L)^3^	65.0 ± 36.6	38.2 ± 15.4	0.355
HsTNT (μg/L)^3^	1.51 ± 1.76	0.0057 ± 0.0064	0.009^∗∗^
Concurrent medications			
ACE inhibitors (%)^1^	79.3%	0	0.000^∗∗^
Beta-blockers (%)^1^	96.6%	0	0.000^∗∗^
Nitrates (%)^1^	96.6%	5.0%	0.000^∗∗^
Statins (%)^1^	100.0%	0	0.000^∗∗^
Aspirins (%)^1^	100.0%	0	0.000^∗∗^
Clopidogrel (%)^1^	61.1%	0	0.001^∗∗^

*BMI, body mass index; DM, diabetes mellitus; SBP, systolic blood pressure; DBP, diastolic blood pressure; TC, total cholesterol; TG, total triglycerid; HDL, high-density lipoprotein; LDL, low-density lipoprotein; WBC, white blood cell; Cr, creatinine; CK-MB, creatine kinase-MB; HsTNT, troponin T. Data are shown as the mean ± SD; ^∗^p < 0.05; ^∗∗^p < 0.01. ^1^Chi-square tests for the differences in the distribution frequencies between AMI patients and controls. ^2^Student’s t-test for the differences between the AMI and control groups. ^3^Mann–Whitney U-test for the differences between the AMI and control groups.*

### The Expression Patterns of Circulating miRNAs by RT-qPCR

**FIGURE 1:**
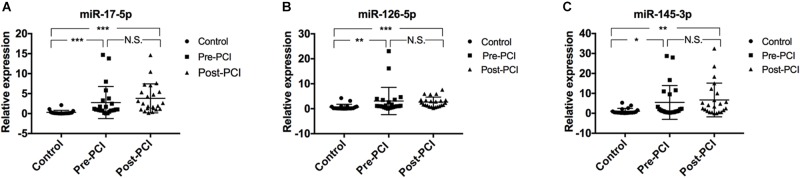
Plasma miRNA levels in the validation population. The scatter plots show the expression levels of **(A)** miR-17-5p, **(B)** miR-126-5p, and **(C)** miR-145-3p measured by quantitative real-time polymerase chain reaction (qRT-PCR) in patients with AMI (including patients before and after PCI operation) (*n* = 29) and control subjects (*n* = 21). The relative miRNA expression levels were normalized to cel-miR-39 and calculated by –ΔΔCt. Differences between each groups were compared by Kruskal-Wallis ANOVA test; ^∗^*p* < 0.05, ^∗∗^*p* < 0.01, ^∗∗∗^*p* < 0.001. Comparisons for which *p* > 0.5 are denoted as N.S. for no significant difference.

In screening experiments, we selected a number of miRNAs that were supposed to be differentially expressed in the plasma of AMI patients based on previous study (Viereck and Thum, 2017). And then we used qRT-PCR assays to investigate the expression patterns of the three selected miRNAs (miR-17-5p, miR-126-5p, and miR-145-3p) in AMI patients before PCI and after PCI. We used Kruskal–Wallis ANOVA test to compare differences between each two groups (pre-PCI vs. CTRL, post-PCI vs. CTRL, and pre-PCI vs. post PCI). The results showed that the expression levels of all three miRNAs were obviously up-regulated in AMI patients compared to control subjects (Figure 1). As shown in Figure 1, the average expression level in AMI patients before PCI of miR-17b-5p (2.747 ± 4.008), miR-126-5p (3.076 ± 5.468), and miR-145-3p (5.459 ± 8.470) was increased compared to miR-17b-5p control (0.296 ± 0.504), miR-126-5p control (0.664 ± 1.103), and miR-145-3p control (1.057 ± 1.411). Likewise, the average expression level in AMI patients after PCI of miR-17b-5p (3.801 ± 3.632), miR-126-5p (2.643 ± 2.003), and miR-145-3p (6.677 ± 8.456) was increased compared to controls. In order to evaluate the effectiveness of PCI, serum HsTNT of patient was determined 1 h before and also 1 h after PCI. In 21 of the 29 (72.4%) patients showed decrease in HsTNT level after PCI reflecting the effectiveness of PCI (Supplementary Table S2).

**FIGURE 2:**
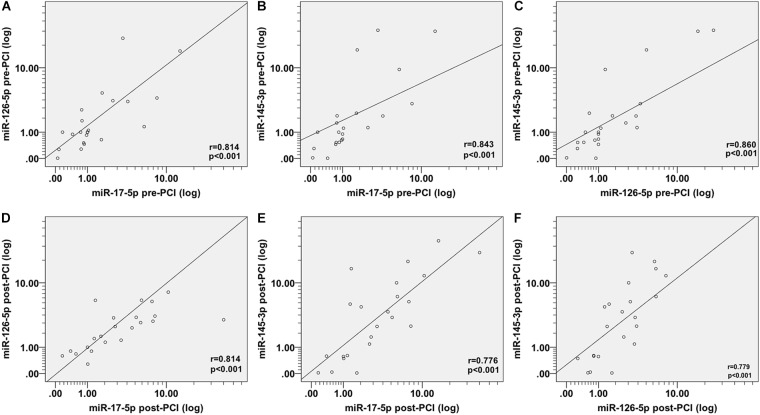
Spearman correlations between the circulating miRNAs in patients with AMI before and after PCI. The scatter plots show the marked correlation in the expression values between **(A)** miR-17-5p before PCI and miR-126-5p before PCI, **(B)** miR-145-3p before PCI, **(C)** between miR-126-5p before PCI and miR-145-3p before PCI, **(D)** between miR-17-5p after PCI and miR-126-5p after PCI, **(E)** miR-145-3p after PCI, and **(F)** between miR-126-5p-after PCI and miR-145-3p-after PCI, in the population (*n* = 29) with AMI.

**FIGURE 3:**
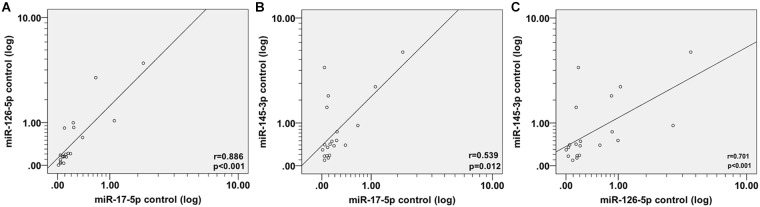
Spearman correlations between circulating miRNAs in control subjects. The scatter plots show the marked correlation in the expression values between **(A)** miR-17-5p and miR-126-5p, **(B)** miR-145-3p, and **(C)** between miR-126-5p and miR-145-3p in the control (*n* = 21) population.

In order to ascertain whether these miRNAs are related to each other or not, we examine the correlation between miRNAs expression using Spearman coefficient analysis. Interestingly, we found a striking correlation between miR-17-5p, miR-126-5p, and miR-145-3p expression in patients before PCI (Figure 2A–C), after PCI (Figure 2D–F) and in the control group (Figure 3A–C).

### The Correlation and Multivariate Analysis

Multivariate analysis between miR-17-5p, miR-126-5p, and miR-145-3p and metabolic parameters or cardiovascular risk factors was performed using Spearman rank correlation coefficients analysis in patients and control subjects (Table 2). In patients after PCI we found a significant negative correlation of miR-145-3p (*r* = −0.615, *p* = 0.009) (Table 2) levels with total triglyceride, whereas no correlation was found in control subjects. A significant negative correlation was found between miR-145-3p (*r* = −0.451, *p* = 0.060) (Table 2) levels with total cholesterol. LDL-cholesterol was significantly correlated with miR-17-5p levels (*r* = −0.476, *p* = 0.046) (Table 2) in patients. A significant positive correlation of miR-17-5p (*r* = 0.557, *p* = 0.011), miR-126-5p (*r* = 0.428, *p* = 0.033), and miR-145-3p (*r* = 0.431, *p* = 0.025) level was found with NT-proBNP before PCI (Table 2). CK-MB was negatively correlated with miR-145-3p (*r* = −0.504, *p* = 0.028) (Table 2).

**Table 2:** Relationships between miR-17-5p, miR-126-5p, miR-145-3p, and classical cardiovascular risk factors in patients with AMI and control subjects.

Variable		AMI before PCI (*n* = 29)			AMI after PCI (*n* = 29)			Controls (*n* = 21)		
		miR-17-5p	miR-126-5p	miR-145-3p	miR-17-5p	miR-126-5p	miR-145-3p	miR-17-5p	miR-126-5p	miR-145-3p
Age (years)	*r* =	0.317	−0.179	−0.182	−0.299	−0.154	0.313	−0.538	−0.669	−0.485
	*p* =	0.141	0.363	0.384	0.213	0.451	0.221	0.015	0.001	0.030
BMI (kg/m^2^)	*r* =	−0.160	−0.210	−0.155	0.105	0.061	0.052	−0.519	−0.392	−0.233
	*p* =	0.456	0.348	0.459	0.624	0.781	0.814	0.019	0.087	0.323
SBP (mmHg)	*r* =	−0.172	−0.110	−0.167	−0.107	−0.056	−0.017	0.158	0.172	−0.020
	*p* =	0.421	0.626	0.415	0.619	0.800	0.939	0.505	0.469	0.932
DBP (mmHg)	*r* =	−0.024	0.164	0.248	0.255	0.078	−0.011	0.480	0.434	0.151
	*p* =	0.910	0.466	0.232	0.229	0.723	0.960	0.032	0.056	0.526
HP (cmp)	*r* =	−0.035	−0.314	−0.096	−0.104	0.004	−0.042	−0.082	0.203	0.526
	*p* =	0.870	0.155	0.649	0.629	0.984	0.849	0.722	0.391	0.017
GLU (mmol/L)	*r* =	0.160	−0.244	−0.146	−0.143	−0.427	−0.526	0.406	0.428	−0.614
	*p* =	0.489	0.211	0.497	0.487	0.033	0.007	0.076	0.067	0.005
Tg (mmol/L)	*r* =	0.400	−0.090	0.240	0.284	−0.400	−0.615	0.099	0.074	−0.232
	*p* =	0.112	0.422	0.247	0.179	0.115	0.009	0.677	0.758	0.326
Tc (mmol/L)	*r* =	−0.213	0.272	0.167	−0.422	−0.330	−0.451	0.158	0.135	0.115
	*p* =	0.277	0.220	0.426	0.092	0.100	0.060	0.518	0.571	0.629
HDL-C (mmol/L)	*r* =	−0.373	−0.378	−0.367	−0.208	−0.213	−0.254	−0.160	−0.277	0.238
	*p* =	0.096	0.075	0.060	0.308	0.296	0.211	0.514	0.237	0.374
LDL-C (mmol/L)	*r* =	−0.279	−0.181	−0.177	−0.476	−0.271	−0.325	0.319	0.216	0.538
	*p* =	0.233	0.386	0.483	0.046	0.181	0.188	0.183	0.390	0.031
WBC (^∗^10ˆ9/L)	*r* =	−0.203	−0.024	0.051	−0.258	−0.354	−0.482	−0.133	−0.087	−0.160
	*p* =	0.353	0.917	0.813	0.235	0.106	0.023	0.567	0.708	0.489
Cr (μmol/l)	*r* =	−0.006	0.237	0.270	0.331	0.081	0.045	−0.385	−0.476	−0.383
	*p* =	0.979	0.301	0.202	0.123	0.719	0.844	0.103	0.040	0.106
NT-proBNP (ng/ml)	*r* =	0.557	0.428	0.431	0.208	0.171	−0.242	−	−	−
	*p* =	0.011	0.033	0.025	0.422	0.528	0.426	−	−	−
CK-MB (μg/l)	*r* =	0.405	−0.153	0.086	−0.294	−0.343	−0.504	−0.377	−0.327	−0.277
	*p* =	0.085	0.484	0.736	0.196	0.118	0.028	0.123	0.171	0.266
hsTNT (μg/l)	*r* =	0.349	0.298	0.267	0.466	−0.212	−0.195	−	−	−
	*p* =	0.143	0.215	0.269	0.025	0.343	0.372	−	−	−

*r = Spearman rank correlation coefficients, p = p-value. BMI, body mass index; DM, diabetes mellitus; SBP, systolic blood pressure; DBP, diastolic blood pressure; TC, total cholesterol; TG, total triglycerid; HDL, high-density lipoprotein; LDL, low-density lipoprotein; WBC, white blood cell; Cr, creatinine; CK-MB, creatine kinase-MB; HsTNT, troponin T.*

**FIGURE 4:**
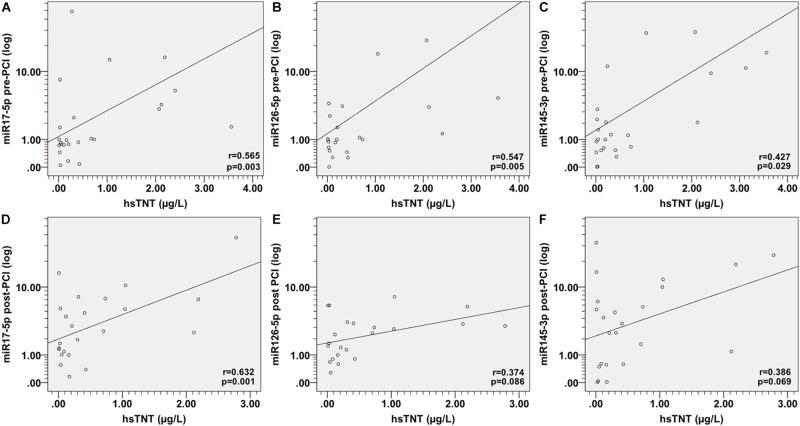
Spearman correlations between circulating miRNAs and HsTNT in AMI patients. The scatter plots show the correlation between HsTNT and **(A)** miR-17-5p pre-PCI, **(B)** miR-126-5p pre-PCI, **(C)** miR-145-3p pre-PCI, **(D)** miR-17-5p post-PCI, **(E)** miR-126-5p post-PCI, and **(F)** miR-145-3p post-PCI, in the population (*n* = 29) with AMI.

We investigated the relationship between HsTNT and miRNAs by Spearman rho correlation coefficient test. The results indicated that the expression levels before PCI of miR-17-5p (*r* = 0.565, *p* = 0.003), miR-126-5p (*r* = 0.547, *p* = 0.005), and miR-145-3p (*r* = 0.427, *p* = 0.029) exhibited a significantly positive correlation with HsTNT, respectively (Figure 4A–C). The expression level of miR-17-5p after PCI was also significantly positively correlate with HsTNT (Figure 4D). However, the expression level of miR126-5p post PCI (Figure 4E) and miR145-3p post PCI (Figure 4F) were not correlated with HsTNT.

### The Diagnostic Accuracy of the Candidate miRNA in AMI

**FIGURE 5:**
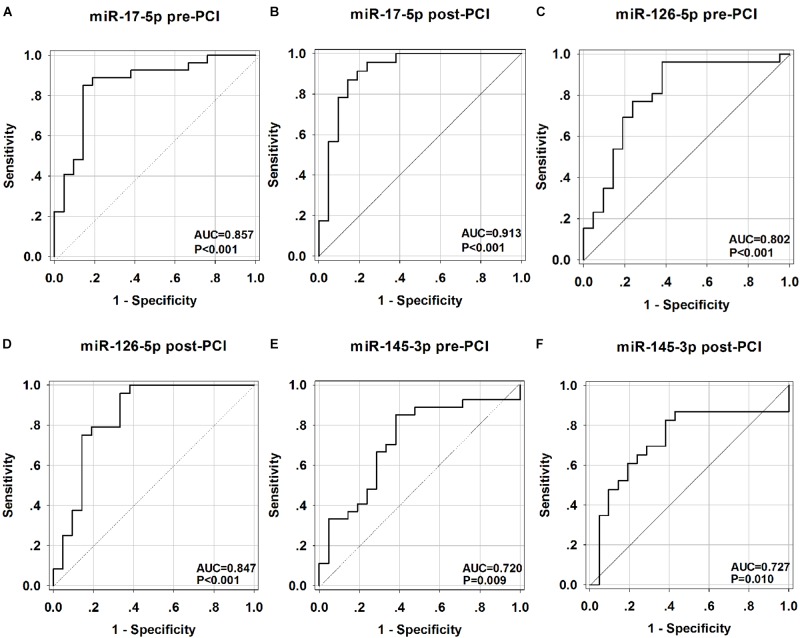
Receiver operating characteristic (ROC) curves analysis of miR-17-5p, miR-126-5p, miR-145-3p for predicting AMI. The areas under the curves (AUC) are 0.857 (95% CI: 0.744–0.965, *p* < 0.001) for miR-17-5p-before PCI **(A)**, 0.913 (95% CI: 0.821–1.005, *p* < 0.001) for miR-17-5p-after PCI **(B)**, 0.802 (95% CI: 0.665–0.936, *p* < 0.001) for miR-126-5p-before PCI **(C)**, 0.847 (95% CI: 0.724–0.971, *p* < 0.001) for miR-126-5p-after PCI **(D)**, 0.720 (95% CI: 0.570–0.870, *p* = 0.010) for miR-145-3p-before PCI **(E)**,0.727 (95% CI: 0.567–0.886, *p* = 0.010) for miR-145-3p-after PCI **(F)**. CI, confidence interval.

**FIGURE 6:**
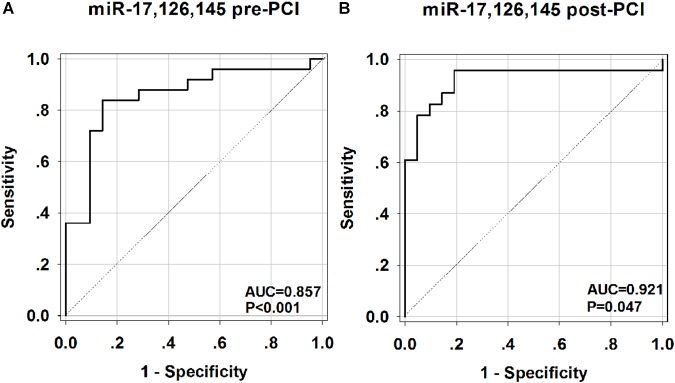
Receiver operating characteristic (ROC) curves analysis of the combination of miR-17-5p, miR-126-5p, miR-145-3p for predicting AMI. The areas under the curves (AUC) are 0.857 (95% CI: 0.743–0.971, *p* < 0.001) for the combination of miR-17-5p, miR-126-5p, miR-145-3p before PCI **(A)** and 0.921 (95% CI: 0.829–1.014, *p* = 0.047) for the combination of miR-17-5p, miR-126-5p, miR-145-3p after PCI **(B)**. CI, confidence interval.

To investigate the accuracy of these circulating miRNA serving as biomarkers for AMI, a ROC curve analysis was performed. As shown in Figure 5, the area under the curve (AUC) before PCI was 0.857 (*p* < 0.001) with cut-off value of 0.709 (85.2% sensitivity, 85.7% specificity) for miR-17-5p (Figure 5A), 0.802 (*p* < 0.001) with cut-off value of 0.619 (100% sensitivity, 61.9% specificity) for miR-126-5p (Figure 5C), 0.720 (*p* = 0.010) with cut-off value of 0.437 (81.8% sensitivity, 61.9% specificity) for miR-145-3p (Figure 5E). We also investigated the AUC value after PCI, which was 0.913 (*p* < 0.001) with cut-off value of 0.727 (87.0% sensitivity, 85.7% specificity) for miR-17-5p (Figure 5B), 0.847 (*p* < 0.001) with cut-off value of 0.625 (95.8% sensitivity, 66.7% specificity) for miR-126-5p (Figure 5D), 0.727 (*p* = 0.010) with cut-off value of 0.445 (82.6% sensitivity, 61.9% specificity) for miR-145-3p (Figure 5F). To further evaluate the effects of these three miRNAs in AMI diagnosis, the combination of miRNA-17-5p, miRNA-126-5p, and miRNA-145-3p resulted in high AUC results of 0.857 (95% CI: 0.743–0.971, *p* < 0.001) with cut-off value of 0.697 (84.0% sensitivity, 85.7% specificity) before PCI (Figure 6A) and 0.921 (95% CI: 0.829–1.014, *p* = 0.047) with cut-off value of 0.767 (95.7% sensitivity, 81.0% specificity) after PCI (Figure 6B).

## Discussion

Recently, an increasing number of circulating miRNAs have been explored as novel candidate biomarkers for diagnostic and prognostic in cardiovascular disease (Fichtlscherer et al., 2010; Viereck and Thum, 2017). It has been mentioned that a variety of physiological and pathological changes occur in the courses of AMI, including endothelial dysfunction, plaque rupture, platelet aggregation, coronary thrombosis, myocardial ischemia, and reperfusion injury (Fuster et al., 2005; Crea and Liuzzo, 2013). Circulating miRNAs are being explored for potential use in diagnosis or prognosis of cardiovascular diseases, because their specific expression pattern, rapid release into the body fluids, and remarkable stability in plasma (Beaumont et al., 2017; Faccini et al., 2017).

In this study, we delineated the temporal expression profile of three miRNAs at the early stage of AMI. By performing ROC curve analysis, we demonstrated that miR-17-5p, miR-126-5p, miR-145-3p may serve as novel candidate diagnostic biomarkers at the early stage of AMI, allowing to distinguish patients with AMI from non-AMI control subjects. The present study showed that the expression levels of circulating miR-17-5p, miR-126-5p, and miR-145-3p were significantly up-regulated in the early phase of AMI. Multivariate analysis showed that high levels of circulating miR-17-5p, miR-126-5p, and miR-145-3p were associated with AMI (before and after PCI). Further analysis confirmed that miR-17-5p, mir-126-5p, and mir-145-3p are related not only to the diagnosis of AMI, but also to synergy between three RNA. The receiver operating curve (ROC) analysis further revealed that miR-17-5p, miR-126-5p, and miR-145-3p may be potential markers for AMI. Interestingly, the combination of the three miRNAs enable to increase the ROC analysis performance with the higher AUC value before and after PCI, indicating that the combination of three miRNAs displayed improved accuracy in diagnosing of AMI.

Among these three miRNAs, miR-17-5p showed the most accurate diagnosing result with the highest AUC value before and after PCI. Furthermore, miR-17-5p exhibited well positive correlation with HsTNT both before and after PCI. These result demonstrated the great value in clinical implication of miR-17-5p. In addition, therapeutic interventions such as statins or anti-platelet agents may influence the expression levels of miRNAs (Weber et al., 2011). Our results showed that even after the use of medication the circulating level of the three miRNAs were still independently associated with AMI, which supporting the significance of these miRNAs in AMI diagnosing. Interestingly, the levels of these three miRNAs were observed increased in the early stage of AMI (within 4 h of onset of symptoms). This result will support the advantages of miRNAs over cardiac troponins, because cTnI was only detected in 85% of patients at this early stage of AMI (Wang G.K. et al., 2010).

There is mounting evidence that apoptosis plays a critical role in myocardial ischemia or reperfusion injury, and contributes to cardiomyocyte loss in the early phase, as well as after myocardial infarction, leading to pathological remodeling, and heart failure (McCully et al., 2004; Abe et al., 2013). Several miRNAs have been implicated in the regulation of pathological and physiological process of heart disease (Ren et al., 2009; Wang X. et al., 2010; Pan et al., 2012). MiR-17-5p is a member of miR-17∼92 cluster, located on the human chromosome 13q31 (Mogilyansky and Rigoutsos, 2013; Zhou et al., 2014). In the previous study, miR-17-5p was demonstrated to be up-regulated under I/R-I and oxidative stress, and could induce apoptotic cell death (Du et al., 2014). Various risk factors can induce EC injury and apoptosis, leading to endothelial dysfunction, which is the initial step in the development of atherosclerosis. Recently, miR-126-5p has been reported to be involved in EC functions by controlling EC activation and leucocyte trafficking (Zhang et al., 2016). Previous studies have demonstrated that miR-145 is predominantly expressed in aortic smooth muscle cells (SMCs) (Cordes et al., 2009; Elia et al., 2009). The similar expression of miR-145 was found in cardiac fibroblasts and SMCs, when cardiac fibroblasts undergo rapid proliferation and transdifferentiation to myofibroblasts (Wang et al., 2014). Although these three miRNAs have been reported in CAD patients with unstable angina (UA) and STEMI before (Zhong et al., 2018), our study is the first time to show the increased miR-17-5p, miR-126-5p, and miR-145-3p were associated with AMI before and after PCI.

## Conclusion

In summary, we investigated the dynamic expressions of circulating miR-17-5p, miR-126-5p, and miR-145-3p in the early phase of AMI (before and after PCI) for the first time. Our results proved that circulating miR-17-5p, miR-126-5p, and miR-145-3p may be considered as novel and promising biomarkers for early diagnosis of AMI by using blood based non-invasive methods. The unique signature of circulating miRNA in AMI patients suggests that plasma miR-17-5p, miR-126-5p, and miR-145-3p may provide useful information to elucidate the mechanism underlying the pathogenesis of AMI.

## Author Contributions

PL and CZ designed the experiments. ZS and LZ collected the blood samples. SX and DL performed the experiments and analyzed the data. SX, DL, PL, and CZ wrote the manuscript. All authors have reviewed and approved the manuscript.

## Conflict of Interest Statement

The authors declare that the research was conducted in the absence of any commercial or financial relationships that could be construed as a potential conflict of interest.
