# Supplementary material for: Circulating MiR-17-5p, MiR-126-5p and MiR-145-3p Are Novel Biomarkers for Diagnosis of Acute Myocardial Infarction
Source: Front Physiol. 2019 Feb 18;10:123. doi: 10.3389/fphys.2019.00123 (PMC6387945; doi:10.3389/fphys.2019.00123)
Supplement: Supplementary file 1 [file Table_1.docx]

**Circulating MiR-17-5p, MiR-126-5p and MiR-145-3p are Novel Biomarkers for Diagnosis of Acute Myocardial Infarction**

Sheng Xue^1^*, Dacheng Liu^2^, Wenjie Zhu^2^, Zhe Su^2^, Liwei Zhang^2^, Changyong Zhou^2^, Peifeng Li^1^

^1^ Institute for Translational Medicine, College of Medicine, Qingdao University, Qingdao, 266021, China.

^2^ Affiliated Hospital of Qingdao University, Qingdao University, Qingdao, 266003 China.

Correspondence and requests of materials should be addressed to S. X. (email: shengxue198@126.com)

**Table S1.** Primer sequences of miRNAs.

| microRNAs | Primer |
| --- | --- |
| miR-17-5p | F Primer: GCGCAAAGTGCTTACAGTGC |
| miR-126-5p | F Primer: GCGCGCATTATTACTTTTGG |
| miR-145-3p | F Primer: GCGTCCAGTTTTCCCAGGA |
| cel-miR-39-3p | F Primer: TCACCGGGTGTAAATCAGCTTG |
| Unified reverse primer | R Primer: TGGTGTCGTGGAGTCG |

**Table S2. Serum HsTNT level of AMI patient before and after PCI.** Serum HsTNT of AMI patient was determined 1 h before and also 1h after PCI. The decrease of HsTNT was indicated in bold. Myocardial damage was defined as HsTNT level ≥ 0.017 µg/L.

| Patient | STEMI/NSTEMI | Serum HsTNT  before PCI (µg/L) | Serum HsTNT  after PCI (µg/L) |
| --- | --- | --- | --- |
| J1 | NSTEMI | **1.040** | **0.919** |
| J2 | STEMI | **0.733** | **0.421** |
| J3 | STEMI | 0.408 | 0.752 |
| J4 | NSTEMI | **0.163** | **0.052** |
| J5 | STEMI | **2.210** | **1.050** |
| J6 | NSTEMI | **0.030** | **0.020** |
| J7 | STEMI | 0.052 | 0.585 |
| J8 | NSTEMI | **0.117** | **0.046** |
| J9 | STEMI | **6.480** | **2.540** |
| J10 | NSTEMI | 0.021 | 0.023 |
| J11 | NSTEMI | **0.107** | **0.017** |
| J12 | NSTEMI | **0.398** | **0.196** |
| J13 | NSTEMI | **0.084** | **0.061** |
| J14 | NSTEMI | **0.210** | **0.150** |
| J15 | NSTEMI | 0.009 | 0.013 |
| J16 | STEMI | 2.070 | 4.280 |
| J17 | NSTEMI | **0.704** | **0.197** |
| J18 | STEMI | **3.630** | **0.429** |
| J19 | NSTEMI | **2.190** | **1.010** |
| J20 | STEMI | **2.120** | **1.042** |
| J21 | STEMI | **0.313** | **0.125** |
| J22 | NSTEMI | **0.699** | **0.274** |
| J23 | STEMI | **1.250** | **0.884** |
| J24 | STEMI | 0.009 | 0.009 |
| J25 | NSTEMI | 0.179 | 3.560 |
| J26 | STEMI | **2.780** | **2.400** |
| J27 | STEMI | **3.170** | **0.037** |
| J28 | STEMI | **0.283** | **0.238** |
| J29 | STEMI | 3.130 | 3.300 |
